# In *E. coli* , the expression of mechanosensitive channels depends on environmental conditions

**DOI:** 10.17912/micropub.biology.001133

**Published:** 2025-07-17

**Authors:** Sarah C Johnson, Sydney Hauth, Saumya Ramanathan, Hannah R Malcolm

**Affiliations:** 1 Chemistry and Biochemistry, University of North Florida, Jacksonville, Florida, United States; 2 Department of Life and Physical Sciences, Fisk University, Nashville, Tennessee, United States

## Abstract

Bacterial mechanosensitive ion channels are essential for cell survival in response to osmotic downshock, these channels rescue bacteria from lethal pressure changes. In *Escherichia coli* ( *E. coli* ), the genome encodes for a single copy of MscL and six MscS superfamily members (MscS, MscK, MscM, YnaI, YbdG, and YbiO). The majority of these have been shown to gate in response to tension. Here, we determined the relative expression using qPCR of all seven genes in non-modified *E. coli* cultured in a variety of different conditions. In all conditions, the highest expressed genes directly correlate with the most frequently observed channels in patch clamp electrophysiology: MscS, MscM, and MscL.

**Figure 1. Relative Expression of Mechanosensitive Genes in E. coli:**
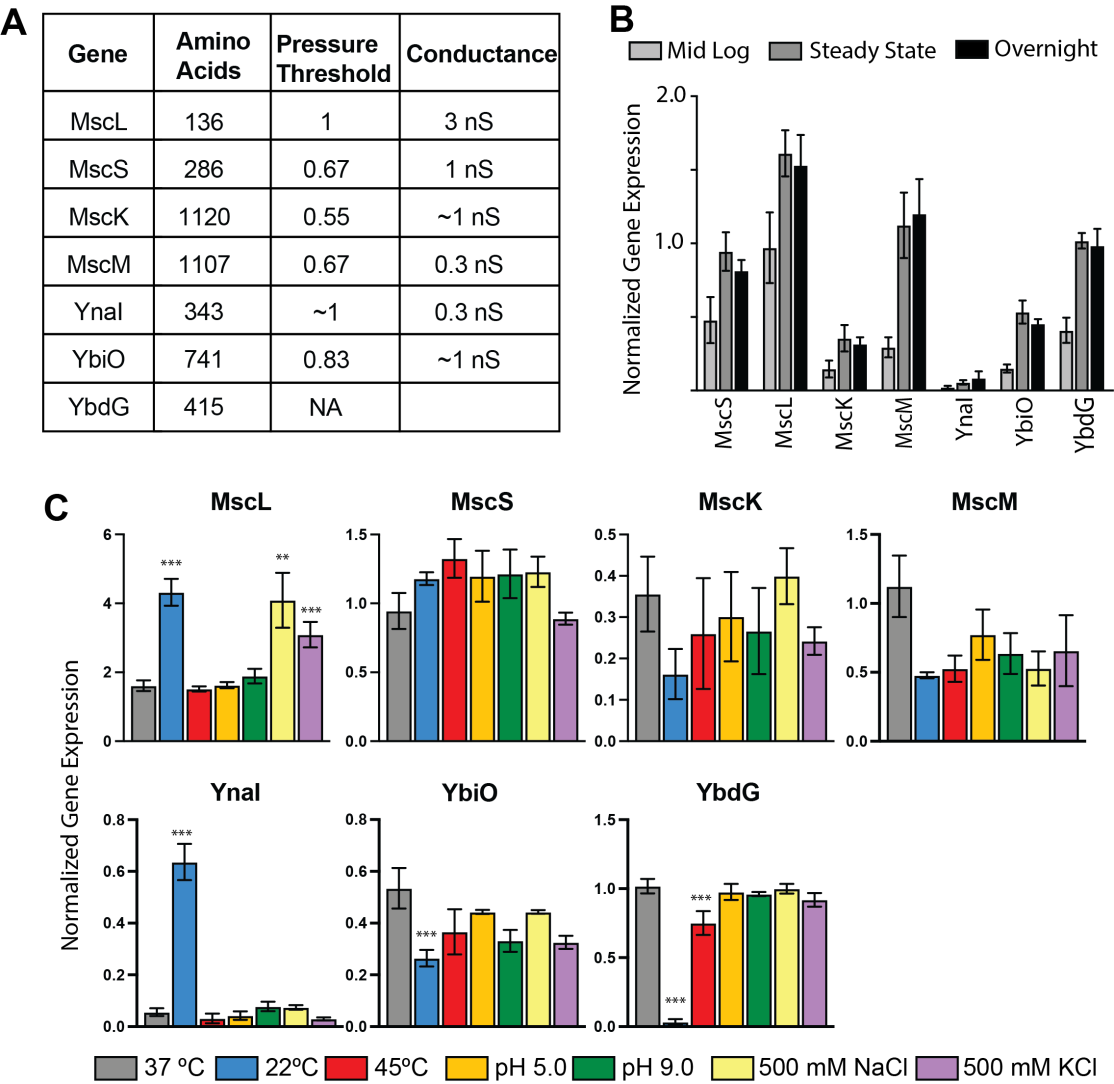
**A)** Table of the seven mechanosensitive genes in *E. coli* reporting their size, pressure threshold relative to MscL (P _X_ /P _L_ ) (Edwards, Black et al. 2012), and their previously reported conductance (Naismith and Booth 2012). **B)** Expression normalized, to RpoB, of the seven E. coli mechanosensitive channels as a function of growth in LB at 37ºC. Error bars represent standard error of the mean, n=4. **C)** The relative expression, to RpoB, for all seven genes under varying growth conditions plotted by gene. (Coloring: LB Lennox at 37ºC (gray), 22ºC (blue), 45ºC (red), pH 5 (golden yellow), pH 9 (green), LB supplemented with 500 mM NaCl (pale yellow) or 500 mM KCl (purple)). The error bars represent the standard error of the mean, for the gray bars n=4, all other remaining samples are n=3. Note that each y-axis is scaled relative to the gene of interest. Stars indicate the statistical significance relative to the same gene in the LB culture as calculated using the Student T-test (**p<0.01, ***p<0.001)

## Description

In bacterial membranes, there are two classes of mechanosensitive channels based on pore size and homology. These two classes were first identified in *Escherichia coli* ( *E. coli* ) membranes, with the mechanosensitive channel of small conductance (MscS) channel opening at ~2/3 the tension of the mechanosensitive channel of large conductance (MscL) channel (Sukharev, Martinac et al. 1993). Further analysis of the *E. coli* genome identified five additional genes that are members of the MscS superfamily, named MscM, MscK, YnaI, YbiO, and YbdG (Li, Moe et al. 2002, Schumann, Edwards et al. 2010, Edwards, Black et al. 2012). Four of these channels (MscM, MscK, YnaI, and YbiO) gate in response to applied tension in patch clamp electrophysiology experiments (Edwards, Black et al. 2012). YbdG has been shown to be involved in hyper-osmotic shock (Schumann, Edwards et al. 2010, Amemiya, Toyoda et al. 2019). These channels have different conductances and variations in the gating tension (Panel A) (Li, Moe et al. 2002, Edwards, Black et al. 2012, Yu, Zhang et al. 2018).

When all seven mechanosensitive channels are deleted from the genome, ~1% of the cells survive osmotic downshock (Edwards, Black et al. 2012). However, when a single channel is knocked out from the genome ~100% of the cells survive osmotic downshock, when MscS, MscL, and MscK are all deleted the survival drops to ~5% (Levina, Totemeyer et al. 1999).Extensive work has been done to characterize the response of these channels to mechanical tension in heterologously expressed channels. However, an in-depth comparative study of the relative expression of all seven channels has not previously been reported (Schumann, Edwards et al. 2010, Bialecka-Fornal, Lee et al. 2012, Edwards, Black et al. 2012). Thus, we explored the relative RNA levels of these genes to determine whether their expression is coupled to specific growth conditions (e.g., high salt, temperature, pH). We hypothesized that all seven genes would show similar expression patterns throughout the growth curve, but that genes directly involved in mechanosensation (such as MscL) would exhibit increased expression under high-salt conditions. We utilized a non-genetically modified *E. coli* strain, ATCC 10798, to understand how these channels are expressed without genomic alterations that could affect expression. We determined the expression, relative to RpoB, for all seven mechanosensitive genes in varying growth conditions. RpoB, an RNA polymerase β-subunit, was selected as the internal standard (Rocha, Santos et al. 2015).

First, we determined if the expression of mechanosensitive genes is growth dependent by isolating RNA throughout the growth curve: mid-log phase, steady-state, and an overnight culture. The relative expression of all seven mechanosensitive genes throughout the growth curve is shown in Panel B. For all mechanosensitive genes the expression at mid-log is less than the expression at steady state or overnight. At steady state, MscL is the highest expressed mechanosensitive channel; MscS, MscM, and YbdG are expressed at similar levels to RpoB. YbiO is expressed at roughly 50% that of MscS, MscK is expressed at ~38% of that of MscS, and YnaI is the least expressed mechanosensitive gene with ~5% of the relative expression of MscS. As the expression of the genes is consistent at both steady state and in an overnight culture, we determined the expression under three different growth conditions at steady state (Panel C). Cells were cultured at different temperatures (22ºC/45ºC), to determine if temperature induced changes in membrane stiffness changed the expression of these channels (Mansilla, Cybulski et al. 2004). We cultured cells in media supplemented in 500 mM NaCl/KCl, to determine if culturing cells in high salt would change the expression of these genes as the cells prepare for possible osmotic downshock. Cells were also cultured in media with different pH (5/9) as the gating kinetics of MscS are known to be altered based on cellular pH (Blount, Sukharev et al. 1999).

The 4 highest expressed channels in the growth curve are highly expressed in all conditions that we tested. MscL shows the highest expression of all channels under all conditions (Panel C). MscL is significantly upregulated at lower temperatures and in the presence of 500 mM salt and shows consistent expression at high temperatures and varying pH. Three members of the MscS superfamily, MscS, MscK and MscM, have consistent expression under all conditions.

The remaining MscS superfamily members show lower expression when compared to MscS, MscM, MscK, and MscL. The major differences in MscS superfamily members, YnaI, YbiO, and YbdG, occur when cells are cultured at varying temperatures. The highest expression of YnaI occurs at 22ºC, under all other conditions minimal expression is observed. YbiO shows slight down-regulation at 22ºC and relatively consistent expression in other conditions. Unlike YnaI and YbiO, YbdG is significantly down regulated at low temperatures and slightly down regulated at higher temperatures. The significant change in expression for MscL, YbiO, and YbdG suggests that these channels could be impacted by changes to the cell membrane at lower temperature.

Cells cultured in varying pHs showed no change in expression for all genes in comparison to the cells cultured at neutral pH, these channels are not pH gated, so changes in expression were not predicted. Cells cultured in high salt, showed a significant upregulation in the expression of MscL, but no significant change in expression for the MscS superfamily members. Recently, Moller et al showed in MJF367 that the expression of MscS is upregulated when MscL is knocked-out (Moller, Britt et al. 2023). This suggests that the expression of these channels may be coupled together. Because MscL has the highest pressure threshold and plays a critical role in survival during osmotic downshock, its upregulation under high-salt conditions indicates that *E. coli* may transcriptionally prime for osmotic stress.

In 2020 Ireland et. al developed Reg-Seq, a new method of characterizing promoters in the *E. coli* genome (Ireland et al., 2020). This study described each of the seven mechanosensitive genes as either activated, repressed, or as having no known regulation. MscM and YbiO were shown to be repressed at their RNA polymerase binding site, MscS was shown to be activated at its RNA polymerase binding site, and MscL, YbdG, and YnaI were shown to have an RNA polymerase binding site but the nature of their regulation was not described. Only MscK was uncharacterized in this study and while the other genes were successfully studied and defined, their promoter was not defined. These regulatory differences suggest that the genes are not transcribed under a shared promoter and likely respond independently to environmental cues.

This work provides a comparative analysis of all seven mechanosensitive genes under diverse environmental conditions. Our data show that MscL, MscS, MscM, and YbdG are consistently the highest expressed channels. We noted that the MscS superfamily members are not up-regulated when cells are cultured in high salt, however MscL is significantly up-regulated. Interestingly, several channels show significant changes in their expression at lower temperatures, these channels have higher pressure thresholds so could be up-regulated to rescue cells. These differences may reflect adaptations in expression to offset shifts in membrane fluidity or osmotic pressure, particularly under stress conditions. Our analysis shows that these seven genes have unique expression profiles at the RNA level, consistent with electrophysiological channel observations. This indicates that these seven genes are non-redundant and each plays an important role in cellular health. Further work exploring the expression, both at the RNA and protein levels, of these genes is required to deeply understand how the expression of these channels is regulated, how the expression of one gene is connected to another, and elucidate how *E. coli* coordinates mechanical stress responses.

## Methods

*Strains and media*

Wildtype E. coli ATCC 10798 was purchased from the American Type Culture Collection (Manassas, VA) and cultured per directives. The LB Lennox media was made according to the manufacturer’s directions and used as the base for all conditions (BD Biosciences, San Jose, CA). Cultures were grown in a MaxQ4000 incubator (ThermoFisher, Waltham, MA) with 220 rpm shaking.

*Growth Curve culture conditions*

In order to collect RNA from different points in the growth curve, an overnight culture, started from a single colony, was grown in LB at 37ºC, 220 rpm. The overnight culture was diluted 1:60 into prewarmed LB. The culture was grown at 37ºC with the optical density at 600 nm (OD600) collected. RNA was collected at mid-log (OD600~ 0.4-0.6), steady-state (OD600~ 1.3), and after ~20 hours of growth (overnight culture). Steady state mRNA was isolated upon no change in OD600 for 60 minutes. For each sample, a 3.6 mL of culture was spun down and used as described below. Four experimental replicates were conducted from unique overnight cultures.

*Growth temperature conditions*

To determine the RNA levels for mechanosensitive channels at different temperatures, an overnight culture was inoculated from a single colony into 3mL LB at 22ºC or 45ºC. The overnight culture was diluted 1:60 into pre-warmed (or pre-cooled) media, when the OD600 of the culture was unchanged for 60 minutes a 3.6 mL culture was collected for RNA isolation. Three experimental replicates were conducted from unique overnight cultures.

*High salt and pH culture conditions*

In order to determine if changes to the media alter the expression of the mechanosensitive channels, media was supplemented with 500 mM NaCl or 500 mM KCl (high salt) or the pH was adjusted to either 5.0 or 9.0 using 6N HCl and 10N NaOH respectively. The media was created by supplementing LB Lennox with the salt or adjusting the pH prior to autoclave sterilization. To determine the RNA levels for mechanosensitive channels under the variable growth conditions, an overnight culture was started from a single colony in 3mL of LB + variable. The overnight culture was diluted 1:60 into prewarmed LB + variable, when the OD600 of the culture was unchanged for 60 minutes a 3.6 mL culture was collected for RNA isolation. Three experimental replicates were conducted from unique overnight cultures for each condition.

*qPCR conditions and Validation*

Total RNA was extracted from all culture conditions using E.Z.N.A. Bacterial RNA Kit (Omega Bio-Tek, R6950, Norcross, GA) using the manufacturer’s standard protocol. 250-300 ng of RNA from every experimental condition was used for a one-step reverse-transcription and quantitative PCR, using BioRad CFX Maestro thermocycler, iTaq Universal SYBR Green One-step Kit (Biorad, 1725151, Hercules, CA). Manufacturer directions were followed for qPCR conditions. Primer sequences for all genes and housekeeping gene, RpoB, are given in the Reagent Table. The expected amplicon sizes were as follows: MscS (348 bp), MscL (224 bp), MscK (456 bp), MscM (432 bp), YnaI (197 bp), YbiO (255 bp), YbdG (282 bp), and RpoB (284 bp). All reactions produced a single melt curve peak, indicating specific amplification. We conducted the analysis of all genes and control for any condition in the same plate to normalize any potential errors.

*Data Analysis*

For panels B and C, we used the comparative threshold cycle (∆Ct) method to quantify the relative expression of each mechanosensitive gene across different growth phases. The Ct value of each target gene was normalized to the housekeeping gene RpoB (∆Ct = Ct _gene_ − Ct _RpoB_ ). Fold change in expression was then calculated as 2 ^(−∆Ct)^ . All data are reported as mean ± standard error of the mean (SEM), and statistical significance was assessed using Student’s t-test.

## Reagents

**Table:** 

MscS qPCR Forward	CGCGCGGATGATTTCCAACGCGGTG
MscS qPCR Reverse	CCGTCTGCAGTACGCATGGTGGTGG
MscL qPCR Forward	GCCGATATCATCATGCCTCCTCTGGG
MscL qPCR Reverse	GCGGCTGCTGGTTCTTCTTTTTTCCG
MscK qPCR Forward	CGGAACGCAATATTAAAGAGCAGATTGCCG
MscK qPCR Reverse	CGACTTAAATTCATCTTTCAGGCTTTGCG
MscM qPCR Forward	CCGAAACTCTCCGCTACTCTGCGCGC
MscM qPCR Reverse	CGCATCCAGTTGCTGGCTCTCTTTTTCCG
YnaI qPCR Forward	GGCGAACATTTCGG
YnaI qPCR Reverse	CCAATTTCCGCTACTGTACC
YbiO qPCR Forward	CCGGCTCCCCGCATAAGCCGTTTAATCCAC
YbiO qPCR Reverse	CCTGGCCGACAAATAATGTCAGGG
YbdG qPCR Forward	GGCTGCAAAAAGGCACCGAAGCG
YbdG qPCR Reverse	CCAACATCAGCACGGCAGCCATTGCACC
RpoB qPCR Forward	GGGCGAAATTCCGCTCATGACAGACAACGG
RpoB qPCR Reverse	GCTCTGTGGTGTAGTTCAGGGCG
	
E.Z.N.A. Bacterial RNA Kit	Omega Bio-Tek, R6950, Norcross, GA
*E. coli* ATCC 10798	ATCC
iTaq Universal SYBR Green One-step Kit	BioRad
